# Direct costs for outpatient excess body weight treatment in Brazilian children and adolescents attending a public children's hospital

**DOI:** 10.1016/j.jped.2024.03.005

**Published:** 2024-04-09

**Authors:** Aline Denise Hanauer, Zaíne Glaci Durte Corrêa, Gleci Blazius, Rodolfo Coelho Prates, Marco Fabio Mastroeni

**Affiliations:** a Universidade da Região de Joinville (UNIVILLE), Programa de Pós-graduação em Saúde e Meio Ambiente, Joinville, SC, Brazil; b Universidade da Região de Joinville (UNIVILLE), Curso de Medicina, Joinville, SC, Brazil; c Universidade da Região de Joinville (UNIVILLE), Curso de Enfermagem, Joinville, SC, Brazil

**Keywords:** Obesity, Overweight, Direct costs, Unified health system, Microcosting

## Abstract

**Objective:**

To estimate the direct costs of treating excess body weight in children and adolescents attending a public children's hospital.

**Methods:**

This study analyzed the costs of the disease within the Brazilian Unified Health System (SUS) for 2,221 patients with excess body weight using a microcosting approach. The costs included operational expenses, consultations, and laboratory and imaging tests obtained from medical records for the period from 2009 to 2019. Healthcare expenses were obtained from the Table of Procedures, Medications, Orthoses/Prostheses, and Special Materials of SUS and from the hospital's finance department.

**Results:**

Medical consultations accounted for 50.6% (R$703,503.00) of the total cost (R$1,388,449.40) of treatment over the period investigated. The cost of treating excess body weight was 11.8 times higher for children aged 5–18 years compared to children aged 2–5 years over the same period. Additionally, the cost of treating obesity was approximately 4.0 and 6.3 times higher than the cost of treating overweight children aged 2–5 and 5–18 years, respectively.

**Conclusion:**

The average annual cost of treating excess body weight was R$138,845.00. Weight status and age influenced the cost of treating this disease, with higher costs being observed for individuals with obesity and children over 5 years of age. Additionally, the important deficit in reimbursement by SUS and the small number of other health professionals highlight the need for restructuring this treatment model to ensure its effectiveness, including a substantial increase in government investment.

## Introduction

Childhood obesity is a global public health challenge of the 21st century.^1^ The estimates for 2035 indicate a rise in the prevalence of obesity among children/adolescents, increasing from 10 to 20% among boys and from 8 to 18% among girls between 2020 and 2035^2^. In Brazil, there are already 6.4 million children with overweight and 3.1 million children with obesity, with an annual increase of approximately 4.4%^2^. Since excess body weight is associated with several other complications and diseases, its rising prevalence has become a global public health issue.^2^

Children and adolescents with obesity are also at increased risk of developing physiological and metabolic changes early in life.^3^ Regardless of weight, body mass index (BMI) and age, people with obesity are exposed to prejudice and stigma, factors that contribute to increased morbidity and mortality.^4^ The combination of these factors has increased the demand for healthcare and the costs destined for the management of obesity.^5^ However, it is still difficult to estimate the costs associated with excess body weight in children, mainly because of the methodological differences across economic evaluation studies.^6^

Health economic evaluation studies are an important tool to support decision-making by health system managers, particularly because of their responsibility to use resources in such a way that they ensure the rights and well-being of society.^7^ In recent years, there has been growing awareness regarding the importance of investigating this topic; however, data and studies are still scarce.

A recent study using data from different countries demonstrated a global economic burden of approximately US$307.72 per capita for obesity and US$190.51 per capita for overweight when compared to eutrophic children.^8^ In the same study, overweight and obesity caused a per capita increase of US$56.52 in non-hospital costs, US$14.27 in outpatient consultations, US$46.38 in medications, and US$1975.06 in hospitalization.^8^

In Brazil, there is a significant lack of information on the cost of treating children and adolescents with obesity, a fact that impairs actions aimed at preventing the rise of obesity. Data from DATASUS revealed a significant increase in healthcare spending of the Brazilian Unified Health System (SUS) on the treatment of obesity in adolescents aged 15–19 years, from R$190,015.83 in 2008 to R$918,664.40 in 2018.^9^ Within this context, the objective of this study was to describe the direct costs of treating excess body weight in children and adolescents attending a public hospital in Joinville, Santa Catarina. The results will be useful to develop strategies to raise awareness regarding the financial impact of obesity on associated comorbidities.

## Methods

This study followed the Consolidated Health Economic Evaluation Reporting Standards (CHEERS) statement.^10^ The study was approved by the Research Ethics Committee of the University of Joinville Region (Protocol number 4.100.353) in accordance with the guidelines laid down in the Declaration of Helsinki.

### Study population

This study describes the direct outpatient costs for children and adolescents aged 2–18 years undergoing treatment for obesity within SUS. The study was conducted at the Dr. Jeser Amarante Faria Children's Hospital (HJAF), city of Joinville, Santa Catarina, the southern region of Brazil. With about 616,000 inhabitants and a Human Development Index of 0.809, Joinville is the largest city in Santa Catarina.^11^ In 2021, the reported prevalence of excess body weight among 6-year-old children was 64.2%, including 30.1% with obesity.^12^

The hospital provides care for children and adolescents aged 0–18 years. It is managed by a non-profit organization and is a referral center that provides secondary and tertiary pediatric healthcare to 25 municipalities in the northern/northeastern regions of Santa Catarina. All care and procedures offered at HJAF are paid for by SUS, with no costs for the user. The amounts paid by HJAF to service providers do not follow the SUS Table of Procedures, Medicines, Orthoses/Prostheses, and Special Materials (SIGTAP)^13^ but are defined by contracts between HJAF managers and service providers. Thus, the direct costs of outpatient care are described based on the values of the SIGTAP table and the values provided by the finance department of HJAF.

Obesity treatment was classified as complete when the medical record upon outpatient discharge stated that the child/adolescent had reached a eutrophic weight status; in the case of non-adherence to treatment, when the patient had been transferred to another health service once he/she had reached 18 years, and/or in the case of failure to return to the outpatient appointment until 2019. Treatment was classified as ongoing when the patient continued under outpatient follow-up after 2019.

### Data collection

Data corresponding to 2221 children and adolescents aged 2–18 years were collected between 1/10/2019 and 30/4/2023 from the electronic records of the digital files of the HJAF. The patients were selected using the Tasy® Hospital Management System. All patients who met the following criteria were included: attending the HJAF outpatient clinic; age between 2 and 18 years; anthropometric data obtained at first consultation; undergoing treatment from 1/1/2009 to 31/12/2019, regardless of whether it was completed or not; attending at least one consultation during the period investigated, and classified as obesity according to code E66 and subcategories of the International Statistical Classification of Diseases and Related Health Problems (ICD-10).^14^

Information on consultations and examinations was obtained using the individual bottom-up microcosting approach throughout the outpatient follow-up of each participant from 2009 to 2019. The following components were included to describe the direct costs of outpatient management of children with excess body weight: consultations, laboratory tests, imaging examinations, and operational costs of the hospital's outpatient clinic. Expenses for medicines were not included because of the difficulty in identifying the dose, dosage regimen, and place of acquisition. In this study, the costs of outpatient treatment for other health conditions were not considered. Since this is a study from the perspective of SUS, direct costs incurred by patients (transport, clothing, food), indirect costs (loss of work capacity and loss of leisure time due to morbidity and early mortality, absenteeism, and reduced productivity), and intangible costs (pain and suffering, value of lost leisure time and benefits) were not included.

The data obtained from the electronic medical records were transferred to a printed form and subsequently to an electronic spreadsheet. The following information was collected: patient identification (medical record number, name, date of birth, parent's names), sex, anthropometric data at the first consultation, number of medical, nutritional, and psychological consultations, obesity-associated comorbidities, and outpatient examinations during the study period. Information on obesity-associated conditions was also obtained based on the ICD-10[14] (Appendix 1).

### Anthropometry

Anthropometric data were obtained from the hospital's Tasy® Hospital Management System. Patients were weighed on a digital scale (Filizola®, model Personal 200, Campo Grande, Brazil) with a capacity of up to 200 kg to the nearest 50 g. The patients were weighed without shoes, coats, jackets, or similar items. Height was measured with a 220-cm wall stadiometer (Tonelli®, model E150A, Criciúma, Brazil) to the nearest 0.1 cm. Weight status was divided into three categories based on the 2006 WHO growth standards for BMI by age and sex: risk of overweight (> *Z*-score + 1SD and ≤ *Z*-score + 2SD), overweight (> *Z*-score + 2SD and ≤ *Z*-score + 3SD), and obesity (> *Z*-score + 3SD) for children aged 2–5 years.^15^ For children aged 5–18 years, the weight status was classified as overweight (> *Z*-score + 1SD and ≤ *Z*-score + 2SD), obesity (> *Z*-score + 2SD and ≤ *Z*-score + 3SD), and severe obesity (> *Z*-score + 3SD).^16^

### Measurement of direct cost items

Information on the costs reimbursed by SUS (consultations, laboratory and imaging tests) was collected from the SIGTAP table[13] and from the hospital records. Amounts paid by HJAF to service providers (consultations, laboratory and imaging tests) and data on outpatient operational costs (electricity, water, telephone, cleaning materials, printing material, medical supplies, salaries for outpatient staff, maintenance, and laundry services) were provided by the hospital's finance department. Operational costs were calculated by the HJAF management using a top-down microcosting approach, with a weighted allocation of services in which a relative value is assigned to each consultation. The operational costs for the years 2009–2010 were not included because of the lack of registration in the HJAF system.

### Evaluation of direct cost items

The direct costs were defined as: 1) costs reimbursed by SUS: resources spent on medical consultations and laboratory and imaging tests (SIGTAP reference table), and 2) healthcare costs paid by the hospital: resources spent on medical consultations plus operational costs and laboratory and imaging tests (provided by the hospital's finance department). Hospital costs were also calculated by participants and included the costs reimbursed by SUS.

For laboratory tests, the hospital pays the equivalent of 1.8 times the SIGTAP table value, without adjustment during the study period. Nurses, nutritionists, and psychologists, professionals who comprise the HJAF multidisciplinary service, are not paid by SUS and do not receive payment per appointment. These professionals are hired by the hospital under the Consolidation of Labor Laws, and the costs related to these services were not included in the sum of resources that made up the direct costs.

To convert the values practiced in the past to current market conditions, annual direct costs were deflated according to the Extended National Consumer Price Index (IPCA) using the cumulative rates in 2019 obtained from the National Consumer Price Index System^17^ (Appendix 2).

### Statistical analysis

Data were stored in a database created in Microsoft® Excel Office and analyzed using the IBM SPSS Statistics 29.0 for Macintosh (Released 2022, IBM Corp., Armonk, New York, USA) and STATA (Statistics Data Analysis, version MP-13.1) programs. Measures of central tendency and dispersion were calculated for quantitative variables and frequency distributions for categorical variables. To examine differences between sexes, the mean hospital costs were compared using the Student *t*-test.

## Results

Among the 2323 patients eligible to participate in the study, 90 were not included because of the lack of anthropometric data at first consultation and 12 because of a BMI Z-score for age and sex ≤1SD. Thus, the final samples consisted of 2221 individuals.

The characteristics of the study participants are presented in Table 1. There was a higher percentage of females (53.5%), patients ≥ 10 years at first consultation (56.6%), participants who had 2–4 medical consultations (38.8%), participants not seen by nutritionists (75.6%) or psychologists (95.6%), and participants with 1–3 types of comorbidities (65.5%) (Table 1). Regarding weight status, 99.8% of the patients were classified as having excess body weight (overweight or obesity).

**Table 1:** Characteristics of the study participants (*n* = 2221). Joinville, Brazil, 2009–2019.

Characteristic	n (%)	Mean (SD)
Gender		
Male	1033 (46.5)	
Female	1188 (53.5)	
Age at first consultation (years)		
< 5	166 (7.5)	
5–10	797 (35.9)	
≥ 10	1258 (56.6)	
Medical consultations		
1	631 (28.4)	
2–4	861 (38.8)	
≥ 5	729 (32.8)	
Nutritional consultations		
0	1679 (75.6)	
1	229 (10.3)	
2	115 (5.2)	
≥ 3	198 (8.9)	
Psychological consultations		
0	2123 (95.6)	
1	36 (1.6)	
2	24 (1.1)	
≥ 3	38 (1.7)	
Comorbidities		
0	427 (26.9)	
1–3	1041 (65.5)	
4–6	119 (7.4)	
≥7	3 (0.2)	
Nutritional status ≤ 5 years		
Normal	0	
Risk of overweight	5 (0.2)	
Overweight	33 (1.5)	
Obesity	129 (5.8)	
Nutritional status ≥ 5 years		
Normal	0	
Overweight	358 (16.1)	
Obesity	963 (43.4)	
Severe obesity	733 (33.0)	
Weight (kg)		59.4 (23.4)
Height (cm)		144.6 (19.0)
BMI (kg/m^2^)		27.3 (5.4)

BMI, body mass index; SD, standard deviation.

Table 2 lists the number of consultations and tests according to the type of cost, already corrected by the 2019 IPCA. Among the 2221 patients investigated, only 542 (24.4%) and 98 (4.4%) received nutritional and psychological care, respectively. Medical consultations corresponded to the main component of the direct outpatient costs, i.e., R$703,503.00 (50.6%) of the total cost of treatment during the period investigated, which was R$1388,449.40. Regarding the remaining 49.4% of the total cost, 34.5% (R$478,071.30) was spent on laboratory tests, and 14.9% (R$206,875.10) on imaging tests over the same period (Table 2).

**Table 2:** Number of consultations and tests according to type of cost (*n* = 2221). Joinville. Brazil. 2009–2019.

				Cost*			
	Participants	Consultations/tests		SUS	Hospital	Hospital operational	Total
Characteristic	n	n	%	R$	R$	R$	R$
*Consultations*							
Medical	2221	9523	84.9	130,544.50	413,304.00	290,199.10	**703,503.00**
Nutritional	542	1445	12.9	–	–	–	
Psychological	98	260	2.2	–	–	–	
**Total**		**11,228**	100.0				
*Laboratory tests*							
Uric acid	146	196	0.5	467.20	838.50		
Antistreptolysin O	38	47	0.1	213.60	384.90		
Antithyroglobulin and antithyroid peroxidase antibodies	206	269	0.7	6220.90	11,194.80		
Calcium	112	145	0.4	356.70	640.10		
HDL cholesterol	1353	3287	8.5	15,520.70	27,857.70		
LDL cholesterol	1323	3217	8.4	15,192.00	27,267.60		
Total cholesterol	1395	3493	9.1	8761.90	15,724.00		
Creatine phosphokinase	45	63	0.2	321.10	645.70		
Cortisol	258	339	0.9	4372.90	7849.90		
Creatinine	586	1062	2.8	2591.80	4651.20		
C3 complement	30	35	0.1	887.80	1594.00		
C4 complement	31	36	0.1	877.90	1576.10		
Somatomedin C (insulin-like growth factor 1)	59	85	0.2	1840.60	3304.60		
Dehydroepiandrosterone sulfate	46	55	0.1	810.70	1455.60		
Estradiol	145	206	0.5	2659.00	4773.10		
Antinuclear antibody	50	68	0.2	1745.80	3134.50		
Follicle-stimulating hormone	216	310	0.8	3113.60	5591.90		
Alkaline phosphatase	42	51	0.1	152.70	274.30		
Gamma-glutamyl transferase	71	100	0.3	506.50	909.10		
Fasting blood glucose	1386	3472	9.0	8637.80	15,501.30		
Glycosylated hemoglobin	239	426	1.1	4129.10	7412.40		
Complete blood count	1107	2226	5.8	12,040.80	21,620.70		
Insulin tolerance test	794	1727	4.5	22,573.30	40,529.80		
Luteinizing hormone	216	315	0.8	3597.00	6456.20		
Microalbuminuria	43	67	0.2	721.00	1294.50		
17-Hydroxyprogesterone	40	48	0.1	652.10	1170.60		
Urinalysis	351	479	1.2	2521.70	4525.40		
C-reactive protein	339	589	1.5	2058.20	3694.60		
Potassium	139	171	0.4	428.20	768.40		
Prolactin	33	39	0.1	538.30	966.30		
Sodium	147	185	0.5	467.30	838.60		
Total testosterone	204	285	0.7	3874.50	6957.70		
Glutamic oxaloacetic transaminase or aspartate aminotransferase	764	1445	3.8	3738.80	6715.00		
Glutamate pyruvate transaminase or alanine aminotransferase	763	1441	3.7	3728.90	6697.20		
Triglycerides	1395	3450	9.0	16,386.20	29,411.10		
Thyroid-stimulating hormone	1378	3616	9.4	43,867.50	78,775.50		
Thyroxine	407	714	1.9	7942.80	14,262.70		
Free thyroxine	1181	2890	7.5	44,991.80	80,791.30		
Urea	416	818	2.1	1858.10	3334.50		
Urine culture	81	98	0.3	748.80	1344.40		
Erythrocyte sedimentation rate	118	150	0.4	565.90	1015.70		
25-Hydroxy vitamin D	337	750	1.9	13,213.00	23,721.10		
Rheumatoid factor (Waaler-Rose test)	45	55	0.1	333.50	598.70		
							
**Total laboratory tests**		**38,520**	100.0				
**Total laboratory cost (R$)**				**266,227.30**	**478,071.30**	**–**	**478,071.30**
*Imaging tests*							
Bone age (radiograph)	553	821	40.4	6776.80	60,652.30		
Chest X-ray	105	119	5.9	1716.90	9704.80		
Electrocardiogram	202	267	13.2	2004.50	10,676.60		
Echocardiogram	201	272	13.4	15,540.20	16,723.00		
MAPA	35	47	2.3	968.10	0.00		
Abdominal ultrasound	261	390	19.2	19,780.70	83,975.50		
Urinary tract ultrasound	32	35	1.7	1148.30	7644.80		
Breast ultrasound	27	37	1.8	1236.50	8232.20		
Thyroid ultrasound	35	42	2.1	1391.80	9265.90		
**Total imaging tests**		**2030**	100.0				
**Total imaging cost (R$)**				**50,563.80**	**206,875.10**		**206,875.10**
**Total**		**40,550**	100.0				**1388.449.40**

SUS, Unified Health System.

*Reference year 2019. Corrected by the Extended National Consumer Price Index (IPCA).

Fifty-two types of tests were requested by doctors, including 43 types of laboratory tests and 9 types of imaging tests. The most frequent laboratory tests were the measurement of thyroid-stimulating hormone (9.4%), total cholesterol (9.1%), and blood glucose (9.0%). The most commonly performed imaging tests were bone age assessment by radiography (40.4%), abdominal ultrasound (19.2%), and electrocardiogram (13.4%) (Table 2).

The total and per capita costs of treating excess body weight according to weight status and sex are described in Tables 3 and 4. The results revealed variations in the number of participants, medical consultations, and per capita cost over the years, regardless of age range and weight status. The cost of treating children aged 5–18 years with excess body weight was R$1279,639.50 between 2009 and 2019 and was approximately 11.8 times higher than the cost of treating children aged 2–5 years over the same period (R$108,897.40) (Table 3, Table 4). Additionally, the cost of treating children with obesity aged 2–5 and 5–18 years was approximately 4.0 and 6.3 times higher than the cost of treating overweight, respectively (Table 3, Table 4). Appendix 3 shows the same data according to year. There was an increase in the costs of treating overweight/obesity between 2009 and 2013 and a subsequent reduction until 2019. Furthermore, the amounts invested by the hospital were about 2/3 higher than those reimbursed by SUS over the years and during the same period, particularly for the treatment of obesity (Table 3 and Appendix 3), i.e., for each R$100.00 paid by the hospital for the treatment of children with excess body weight, SUS reimbursed only about R$30.00.

**Table 3:** Total and per capita cost of treating excess body weight in children aged 2–5 years according to weight status and sex (*n* = 167). Joinville, Brazil, 2009–2019.

						Cost^a^					
						Reimbursement by SUS		Hospital cost^b^		Hospital cost by sex	
		Participants		Medical consultations		By participant	Total	By participant	Total	M	F
Risk of overweight/overweight (*n* = 38)	Year	M	F	M	F	R$	R$	R$	R$	R$	R$
	2009	–	–	–	–	–	–	–	–	–	–
	2010	–	4	–	5	67.90	271.50	222.90	891.50	891.50	–
	2011	3	7	7	12	100.60	1005.70	345.20	3452.30	1070.73	2381.57
	2012	2	3	2	4	41.10	205.60	152.50	762.30	222.53	539.77
	2013	2	4	2	4	52.40	314.20	148.60	891.60	153.67	737.93
	2014	1	2	1	2	108.40	325.30	278.60	835.90	74.29	761.61
	2015	4	5	8	9	120.60	1085.30	324.60	2921.50	1157.85	1763.65
	2016	5	5	9	10	104.10	1041.40	302.50	3024.90	1190.82	1834.08
	2017	3	7	8	14	103.10	1030.80	309.00	3090.20	800.50	2289.70
	2018	4	13	11	20	72.10	1226.60	226.30	3847.80	1176.44	2671.36
	2019	4	8	6	13	52.20	625.90	171.90	2063.30	671.13	1392.17
Total risk of overweight/overweight		28	58	54	93		7132.30		21,781.30	7409.43	14,371.87
Mean										740.94	1596.88^c^
Standard deviation										442.68	784.96
Obesity (*n* = 129)											
	2009	2	1	3	1	24.50	73.60	77.60	232.90	174.68	58.22
	2010	11	5	15	11	92.40	1478.00	209.80	3357.50	2086.96	1270.54
	2011	12	13	22	28	87.20	2181.10	298.10	7452.80	3263.65	4189.15
	2012	12	15	29	37	101.40	2736.70	325.90	8799.00	3578.64	5220.36
	2013	17	17	24	37	101.70	3457.50	279.80	9513.90	3834.33	5679.57
	2014	17	17	29	35	82.50	2803.80	254.40	8650.80	3896.41	4754.39
	2015	17	13	32	30	132.80	3984.90	394.70	11,842.10	5663.35	6178.75
	2016	19	12	35	20	98.90	3064.50	286.60	8885.60	5052.81	3832.79
	2017	23	20	52	33	76.30	3280.70	234.00	10,061.10	6236.29	3824.81
	2018	21	14	42	25	91.00	3184.50	272.70	9546.10	5351.36	4194.74
	2019	16	18	36	34	83.60	2840.90	258.10	8774.30	4588.61	4185.69
Total obesity		167	145	319	291		29,086.20		87,116.10	43,727.09	43,389.01
Mean										3975.19	3944.45
Standard deviation										1734.72	1809.60
Total overweight/obesity		195	203	373	384		36,218.50		108,897.40	51,136.52	57,760.88

M, male; F, female; SUS, Unified Health System.

a Reference year 2019. Corrected by the Extended National Consumer Price Index (IPCA).

b Including reimbursement by SUS.

c Student *t*-test between sexes (*p* < 0.05).

**Table 4:** Total and per capita cost of treating excess body weight in children aged 5–18 years according to weight status and sex (*n* = 2054). Joinville, Brazil, 2009–2019.

						Cost^a^					
						Reimbursement by SUS		Hospital cost^b^		Hospital cost by sex	
		Participants		Medical consultations		By participant	Total	By participant	Total	M	F
Overweight (*n* = 358)	Year	M	F	M	F	R$	R$	R$	R$	R$	R$
	2009	7	10	13	13	47.10	800.90	120.00	2040.40	897.54	1142.86
	2010	28	46	67	81	94.70	7005.20	253.70	18,775.20	7659.04	11,116.16
	2011	27	68	69	144	79.60	7559.50	311.10	29,558.40	9763.76	19,794.64
	2012	29	57	55	85	67.20	5775.80	231.60	19,915.10	7322.77	12,592.33
	2013	26	59	57	121	100.20	8519.50	321.30	27,311.50	10,036.09	17,275.41
	2014	24	55	50	97	84.30	6662.70	273.50	21,607.60	7770.73	13,836.87
	2015	22	45	30	90	91.50	6133.40	287.00	19,229.50	4845.98	14,383.52
	2016	21	36	34	59	68.70	3915.40	228.80	13,039.10	4166.50	8872.60
	2017	12	34	20	67	74.60	3430.90	241.30	11,098.50	2475.61	8622.89
	2018	8	28	11	41	64.00	2304.40	204.50	7363.10	1912.59	5450.51
	2019	11	22	20	28	36.70	1210.30	142.40	4700.00	1976.98	2723.02
Total overweight		215	460	426	826		53,318.00		174,638.40	58,827.59	115,810.81
Mean										5347.96	10,528.25^c^
Standard deviation										3308.44	5856.35
Obesity (*n* = 963)											
	2009	23	9	27	13	50.30	1609.68	127.99	4095.57	3338.67	756.90
	2010	55	69	131	138	112.10	13,900.23	298.46	37,008.49	18,834.34	18,174.15
	2011	80	122	186	247	105.08	21,226.72	365.97	73,926.38	30,500.49	43,425.87
	2012	90	122	209	268	109.00	23,107.22	351.55	74,529.26	31,080.41	43,448.85
	2013	100	151	225	302	106.99	26,854.98	335.37	84,176.95	35,430.26	48,746.69
	2014	90	118	180	206	83.86	17,443.43	266.49	55,430.04	26,098.75	29,331.29
	2015	92	125	199	249	98.93	21,467.01	313.40	68,007.05	29,430.62	38,576.43
	2016	90	116	154	181	77.18	15,899.59	242.31	49,915.12	22,035.67	27,879.45
	2017	78	135	152	272	84,46	17,989.76	254.96	54,305.45	19,074.16	35,231.29
	2018	78	128	129	257	87.79	18,084.96	268.40	55,291.34	18,227.75	37,063.57
	2019	58	98	114	190	81.16	12,661.06	263.15	41,052.00	13,553.05	27,498.95
Total obesity		834	1193	1706	2323		190,244.64		597,737.64	247,604.20	350,133.44
Mean										22,509.47	31,830.31
Standard deviation										9255.55	13,537.86
Severe obesity (*n* = 733)											
	2009	13	8	17	8	66.97	1406.41	151.84	3188.64	2157.81	1030.83
	2010	66	42	152	74	125.34	13,536.81	301.38	32,549.30	21,470.48	11,078.82
	2011	108	61	251	120	110.39	18,656.72	362.99	61,344.47	39,208.89	22,135.58
	2012	91	66	231	150	116.14	18,234.05	360.70	56,629.27	34,744.78	21,884.49
	2013	110	76	229	181	105.80	19,678.26	322.79	60,039.64	30,167.37	29,872.27
	2014	95	62	203	128	96.62	15,169.33	305.12	47,904.32	29,326.20	18,578.12
	2015	99	53	227	118	116.85	17,761.70	356.10	54,127.38	34,364.92	19,762.46
	2016	93	73	175	144	94.80	15,736.47	289.88	48,120.80	25,163.83	22,956.97
	2017	112	78	256	179	104.43	19,841.41	307.87	58,495.74	35,688.02	22,807.72
	2018	110	69	229	135	93.96	16,817.97	275.83	49,374.42	31,598.67	17,775.75
	2019	84	52	174	104	78.79	10,716.08	260.95	35,489.39	23,158.70	12,330.69
Total severe obesity		981	640	2144	1341		167,555.22		507,263.38	307,049.70	200,213.68
Mean										27,913.60	18,201.24^c^
Standard deviation										10,158.78	7693.31
Total obesity/severe obesity		1815	1833	3850	3664		357,799.86		1105.001.02	554,653.90	550,347.12
Total overweight/obesity		2030	2293	4276	4490		411,117.86		1279.639.42	613,481.49	666,157,93

M, male; F, female; SUS, Unified Health System.

a Reference year 2019. Corrected by the Extended National Consumer Price Index (IPCA).

b Including reimbursement by SUS.

c Student *t*-test between sexes (*p* < 0.05).

Regarding sex, the mean cost of treating children aged 2–5 years at risk of overweight/overweight was 46.4% higher for females compared to males (R$740.94 [SD = 442.68] vs. 1596.88 [SD = 784.96] for males and females, respectively; *p* < 0.05) (Table 3). A similar (50.8%) result was observed for children overweight aged 5–18 years (R$5347.96 [SD = 3308.44] vs. 10,528.25 [SD = 5856.35] for males and females, respectively; *p* < 0.05) (Table 4). However, the mean cost of treating children with severe obesity aged 5–18 years was 53.4% higher for males compared to females (R$27,913.60 [SD = 10,158.78] vs. 18,201.24 [SD = 7693.31] for males and females, respectively; *p* < 0.05) (Table 4).

## Discussion

In the present study, the cost of treating 2221 Brazilian children and adolescents with excess body weight over the period from 2009 to 2019 was R$1388,449.40, with medical consultations accounting for half of the expenses. The cost of treating obesity was approximately six times higher than the cost associated with treating overweight. Additionally, the mean cost of treating overweight was about 50% higher in females when compared to males. The amount invested by the hospital for the obesity treatment was higher than that reimbursed by SUS over the years.

The present study's data are in line with other studies that found higher amounts being spent on patients with obesity when compared to overweight[8] and eutrophic individuals.^18^, ^19^, ^20^ ​An important finding of this study was the low frequency of consultations with psychologists and nutritionists. Although the treatment of obesity requires the involvement of professionals from different areas,^21^ SUS does not prioritize and does not pay for consultations with any professional other than a general practitioner. In fact, the psychologists/nutritionists in the present study were invited to participate in the treatment of overweight by the doctor responsible for the outpatient clinic but did not receive any type of remuneration.

Regarding sex, the highest mean cost of treating female children with overweight observed in the present study was due to the larger number of medical consultations, about 65% compared to males. In fact, the prevalence of overweight was in general 46–70% higher among female participants than among males.

A deficit in the reimbursement of the hospital by SUS for the treatment of obesity is another important finding that limits the success of treatment by not permitting to hiring other health professionals or improving the physical structure and equipment for patient monitoring.

Despite some successful interventions to prevent obesity in early childhood, the lack of economic health assessments continues to be an important obstacle to controlling its increase.^22^

Studies that perform economic analyses of disease treatment and that address direct costs from the perspective of service providers would be important for developing programs aimed at preventing the onset of excess body weight already in the first years of life. The inadequate organization of hospital-generated data is the main obstacle to gathering information. Data stored in different software that are incompatible and need for printing or transfer to another software so that they can be analyzed is an unacceptable situation given current technological advances. Furthermore, the different economic evaluation methods used across studies make data analysis and comparison even more difficult. The majority of studies are conducted in developed countries that use different healthcare models and mainly address preventive interventions.^6^^,^^23^^,^^24^ Finally, although this study did not evaluate children without excess body weight, it is important to highlight that children with obesity incur additional costs due to the specific consultations/exams related to the treatment of this condition, which contributes to an increase in costs compared to children who are not obese. The additional costs are related to more frequent medical appointments, specific exams, nutritional interventions, or other treatments associated with obesity complications.

This study has some strengths. First, this study used primary data, thus reducing bias when compared to data obtained from public databases. Second, the microcosting approach was used for data analysis, with SUS as the service provider. This method is considered the gold standard for investigating healthcare costs.^25^ Third, the study utilized economic data focusing on the outpatient treatment of obesity and not only on the costs of the disease. This approach permits theidentification of whether the resources are being invested correctly and whether they are successful in reducing excess body weight over the years. Lastly, this is the first study that performed a cost analysis of treating excess body weight in Brazilian children and adolescents using public data, a fact that will enable the development of actions aimed at minimizing costs and maximizing treatment of this disease.

Nonetheless, this study also has some limitations. First, the study provides a partial economic assessment of the direct costs of outpatient obesity treatment by addressing the perspective of the healthcare provider and describing only part of the economic burden of obesity in childhood and adolescence. The financial burden not evaluated in this study refers to direct non-medical costs such as the transportation of children and caregivers, food, physical activity, and indirect costs, including the inability to work or loss of productivity of those involved in managing this disease. Second, the operational costs for 2009–2010 were not available in the data recording system of the hospital where the study was conducted, underestimating part of the values reported in this study. Finally, the inaccurate recording of some information described in the medical records may have limited the interpretation of some data used in the study.

## Conclusion

In conclusion, the costs of treating obesity were found to be higher than those of treating overweight. The findings also highlight the important deficit in reimbursement by SUS compared to the hospital's outpatient costs and the small number of other health professionals working on the team, such as nutritionists and psychologists. Finally, there is a knowledge gap in the cost-effectiveness of treating obesity in Brazil, highlighting the need for restructuring the treatment model to ensure its effectiveness.

## Conflicts of interest

The authors declare no conflicts of interest.

## Appendix Supplementary materials

Image, application 1

## Footnotes

Supplementary material associated with this article can be found in the online version at doi:10.1016/j.jped.2024.03.005.
