# Supplementary material for: Direct costs for outpatient excess body weight treatment in Brazilian children and adolescents attending a public children's hospital
Source: J Pediatr (Rio J). 2024 Apr 9;100(4):444–54. doi: 10.1016/j.jped.2024.03.005 (PMC11331229; doi:10.1016/j.jped.2024.03.005)
Supplement: Supplementary file 1 [file mmc1.docx]

**JPED-D-23-00493_Supplementary Material**

**Appendix 1** Obesity-associated conditions according to the International Statistical Classification of Diseases and Related Health Problems (ICD-10).

E03: Other hypothyroidism and its subcategories.

E10: Insulin-dependent diabetes mellitus.

E23: Pituitary hypofunction and disorders.

E24: Cushing’s syndrome.

E28.2: Polycystic ovary syndrome.

E78: Disorders of lipoprotein metabolism and other lipidemias and its subcategories.

E88: Other metabolic disorders.

G47: Sleep disorders and its subcategories.

I10: Essential hypertension and its subcategories.

I15: Secondary hypertension and its subcategories.

K76: Other liver diseases and its subcategories.

K80: Cholelithiasis and its subcategories.

L83: Acanthosis nigricans.

M21.0: Valgus deformity, not elsewhere classified.

M92.5: Juvenile osteochondrosis of the tibia.

N62: Hypertrophy of breast and its subcategories.

N64: Other disorders of breast and its subcategories.

R45: Symptoms and signs involving emotional state and its subcategories.

R73: Elevated blood glucose level and its subcategories.

Z00: General examination and investigation of persons without complaint and its subcategories.

**Appendix 2** Cumulative Extended National Consumer Price Index (IPCA)* over the period from 2009-2019.

| Period | IPCA |
| --- | --- |
| 2009 - 2019 | 0.8387 |
| 2010 - 2019 | 0.7627 |
| 2011 - 2019 | 0.6644 |
| 2012 - 2019 | 0.5628 |
| 2013 - 2019 | 0.4765 |
| 2014 - 2019 | 0.3941 |
| 2015 - 2019 | 0.3102 |
| 2016 - 2019 | 0.1838 |
| 2017 - 2019 | 0.1141 |
| 2018 - 2019 | 0.0822 |
| 2019 | 0.0431 |

*https://www.ibge.gov.br/estatisticas/economicas/precos-e-custos/9256-indice-nacional-de-precos-ao-consumidor-amplo.html

**Appendix 3** Total cost of treating excess body weight in children aged 5-18 years according to weight status and amount invested by institution. Joinville, Brazil, 2009-2019.
